# Supplementary figures and images for: Piezo1 mechanosensing regulates integrin-dependent chemotactic migration in human T cells
Source: eLife. 2024 Feb 23;12:RP91903. doi: 10.7554/eLife.91903 (PMC10942591; doi:10.7554/eLife.91903)

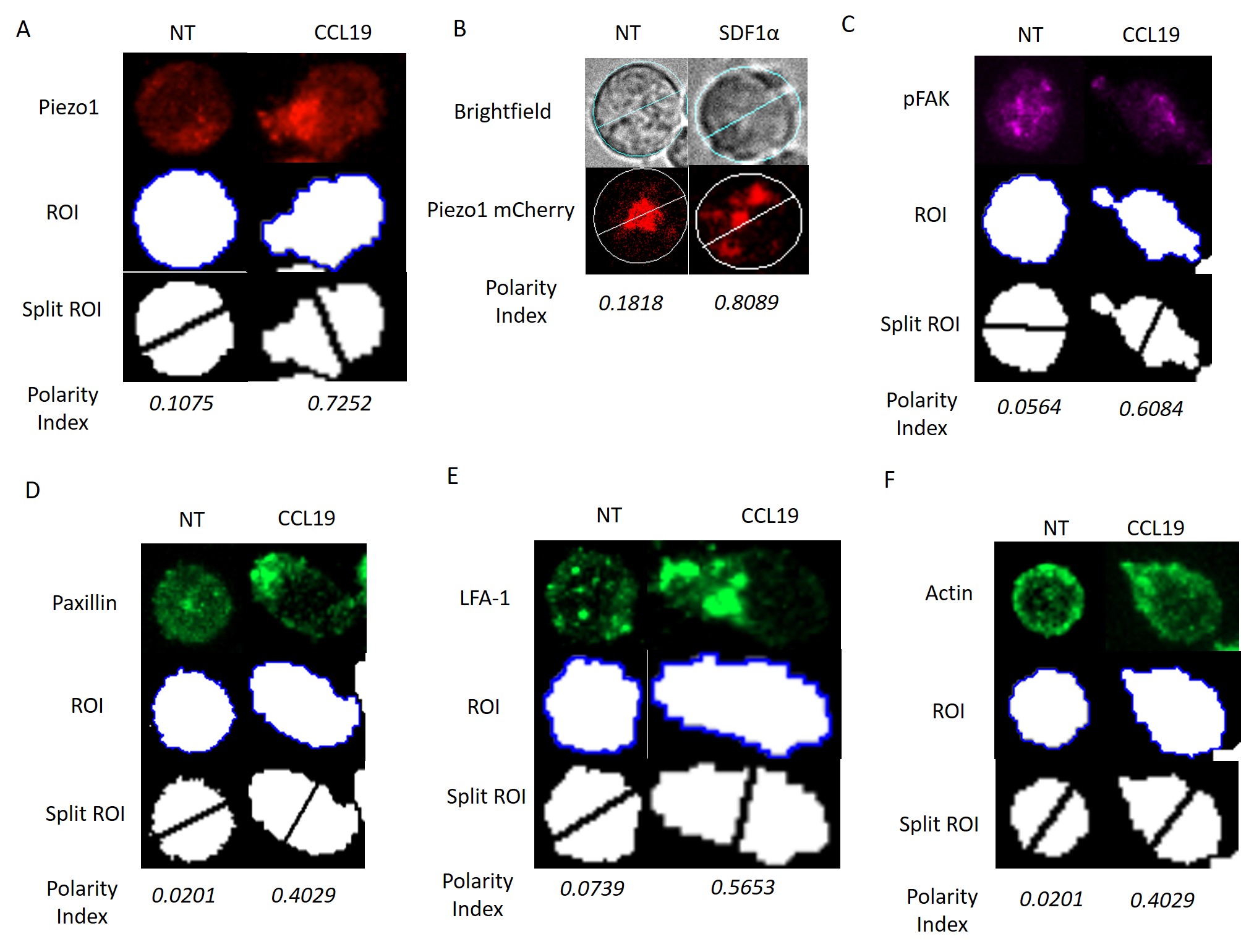

Supplement: Supplementary file 1. — Region-of-interest identifying cell boundary and polarity indices mentioned. All analyses were performed on Fiji (see methods). [file elife-91903-supp1.zip › Supplementary File 2.jpg]
